# Influence of alteration of foot progression angle on three-dimensional pelvic kinematics in individuals with knee osteoarthritis during walking: A randomized crossover study

**DOI:** 10.1097/MD.0000000000048344

**Published:** 2026-04-10

**Authors:** Yongwook Kim

**Affiliations:** a Department of Physical Therapy, College of Medical Sciences, Jeonju University, Jeonju, Jeollabuk-do, South Korea.

**Keywords:** foot position, knee, motion analysis, osteoarthritis, pelvic, segment motion, walking

## Abstract

**Background:**

Foot progression angle (FPA) modification trials during walking have been used to reduce knee adduction moments in medial compartment knee osteoarthritis. However, few studies have confirmed the impact of FPA modification as a clinical intervention on pelvic segment kinematics.

**Methods:**

Kinematic pelvic data were obtained from 37 participants with medial knee osteoarthritis using a three-dimensional motion capture system under varying FPA walking conditions. A repeated-measures analysis of variance was conducted to assess the influence of each FPA condition on kinematic values.

**Results:**

During maximal internal foot rotation gait trials, participants exhibited decreased depression, reduced external rotation, and increased internal rotation of the pelvis compared to the free walking condition (*P* *<* .05). In contrast, under the maximal external foot rotation condition, significantly less internal rotation and increased external rotation of the pelvis were observed compared to the free walking condition (*P* *<* .05).

**Conclusion:**

The findings of this study suggest that additional musculoskeletal dysfunctions and clinical implications, including back pain and pelvic instability, should be considered when implementing FPA modification gait in individuals with medial compartment knee osteoarthritis.

## 1. Introduction

Knee osteoarthritis (OA), characterized by degenerative changes in the knee joint, is highly prevalent among the aging population.^[1–3]^ Approximately one-third of individuals over the age of 60 experience knee OA, a figure that is increasing due to the recent extension of the average lifespan in Korea.^[4]^ Traditionally, knee OA is associated with various deformities due to the degeneration of joint cartilage and surrounding soft tissues; specifically, the occurrence of genu varus deformity is 3.5 times more frequent than that of genu valgus.^[5,6]^ Genu varus deformity worsens knee deformation by increasing the peak knee adduction moment (KAM) and the compressive knee contact force in the medial compartment during the stance phase of walking.^[7–9]^ Knee joint moments observed in real-time during the stance phase of gait (when body weight is supported on the ground while walking) are biomechanical variables calculated by multiplying the magnitude of the three-dimensional (3D) ground reaction force by the lever arm distance from the knee joint center.^[4]^

Among these moments, the peak KAM is correlated with the severity of clinical manifestations, pain, and the degree of deformity in medial knee OA.^[10–13]^ Hence, nonsurgical interventions for medial compartment knee OA with genu varus deformity focus on reducing peak KAM.^[14]^ The KAM exhibits 2 pronounced peak values in the coronal motion plane during gait; the first occurs early and the second late in the stance phase.^[4,8]^ Various gait strategies, including pain-avoidant walking and adjustments to the foot progression angle (FPA), have been implemented to mitigate the 2 peak KAMs in patients with medial knee OA.^[15,16]^

Following the initial study by Wang et al,^[17]^ numerous studies have investigated the biomechanical effects on lower extremity joints in response to FPA adjustments during walking. Not all knee OA patients experience a significant reduction in peak KAM due to FPA modification,^[6]^ yet, on average, a toe-in gait reduces the first peak KAM, and a toe-out gait diminishes the second peak KAM.^[4,15,16,18]^

Despite numerous studies asserting that toe-in or toe-out gait interventions decrease both first and second peak KAMs in patients with medial compartment knee OA, the extent of peak KAM alteration greatly varies among individuals due to factors such as target FPA achievement, anatomical diversity, and the biomechanical properties of the motor link.^[19]^

Therefore, controlling external variables that adversely influence measurements and accurately assessing the peak KAMs in response to FPA changes during gait are critical for securing beneficial clinical outcomes in knee OA patients. Moreover, evaluating the impact of FPA-modified gait training on the biomechanical variables of lower extremity joints and segments using trustworthy and validated methods, such as 3D gait analysis, is essential to assess the presence and severity of knee OA and to forecast the prognosis.^[4,8]^

Previous studies examining the biomechanical effects of FPA-modified gait interventions in individuals with knee OA have primarily focused on their influence on KAM peaks and the kinematic values of the hip, knee, and ankle joints.^[20–22]^ However, investigations into the 3D kinematics of the pelvic segment during ambulation in patients with medial compartment knee OA have been limited. Medial compartment knee OA is generally known to increase the risk of developing low back pain (LBP), pelvic instability, and hip OA. This is due to the shared risk factors associated with musculoskeletal issues in other joints and areas of the lower extremity.^[23–25]^ Thus, the objective of this study was to explore the effects of 3 distinct FPA-modified gait interventions on 3D kinematic pelvic motion in patients with medial compartment knee OA, using reliable and objective 3D motion analysis equipment.

## 2. Methods

### 2.1. Participants

**Figure 1.**
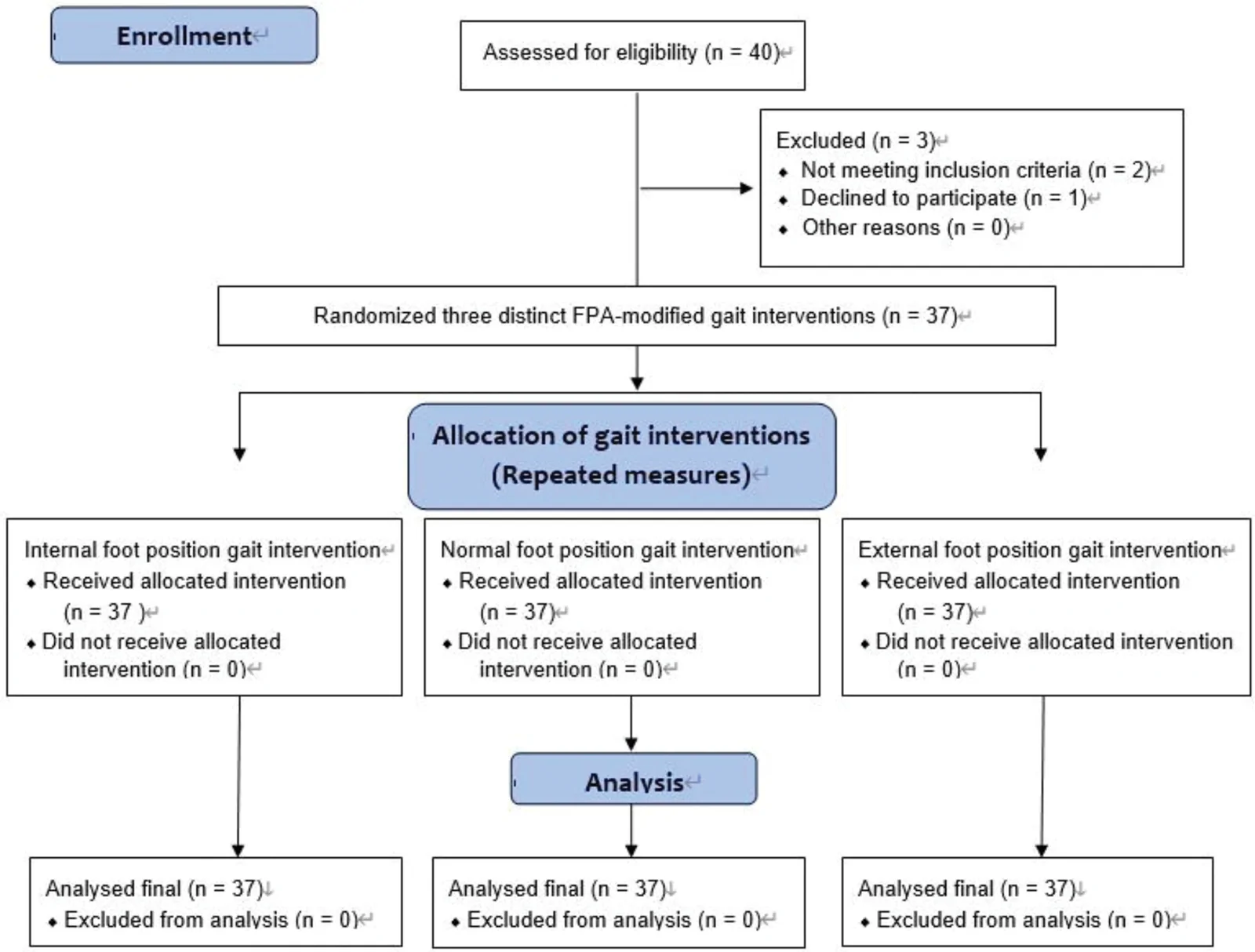
Flow chart of the data selection and analysis process.

Forty patients diagnosed with medial compartment knee OA participated in this study, but 37 individuals ultimately took part because 2 did not meet the inclusion criteria (n = 2) and 1 refused to participate (n = 1) (Fig. 1).

All participants had both knee OA and genu varum deformity but did not experience knee pain during walking. They were recruited from a local orthopedic hospital and managed nonoperatively within the past 5 years. The inclusion criteria were: ability to walk independently without the use of ambulation aids; no surgical history involving lower extremity joints or segments; absence of severe pain in the lower extremity joints or segments that interferes with independent walking; and a Kellgren–Lawrence grade of 3 or lower.^[4]^ Exclusion criteria for study participation included patients with a Kellgren–Lawrence grade ≥4, indicating severe medial compartment knee osteoarthritis, which reduces the effect of FPA modification during walking. Additionally, patients with systemic rheumatoid arthritis, knee pain from other diseases, or pathological gait disturbances were excluded.

The average Kellgren–Lawrence grade among participants was 1.8 ± 0.6 (Grade I: n = 12, Grade II: n = 24, Grade III: n = 1). Participants had an average age of 63.4 years, a height of 161.3 cm, and a weight of 64.0 kg. The average walking velocities during trials in free foot position (FFP), maximal internal foot position (MIFP), and maximal external foot position (MEFP) were 1.21 ± 0.18 m/s, 1.14 ± 0.18 m/s, and 1.19 ± 0.22 m/s, respectively. Additionally, the mean FPA angles during gait trials were 16.99 ± 7.80 degrees for FFP, ‐11.37 ± 6.08 degrees for MIFP, and 44.64 ± 7.23 degrees for MEFP. A research expert thoroughly explained all experimental procedures to the participants who volunteered for the study. Written informed consent was obtained from all participants. The study design and experimental protocol received approval from the Institutional Review Board of Jeonju University (jjIRB-200714-HR-2020-0708).

### 2.2. Instrumentation for pelvic kinematics and data collection

3D pelvic kinematic data during gait were collected in the motion analysis laboratory at Jeonju University. This laboratory was equipped with a 3D motion capture system (Vicon Inc., Oxford, England) that included 8 infrared cameras, 2 force platforms (AMTI, Watertown), and a 10 m walking pathway. The motion capture cameras operated at a sampling rate of 100 Hz, while the force platforms, used for precise analysis of the stance and swing phases during walking, operated at a sampling rate of 500 Hz. A 7.5 cm T-frame wand was utilized as a calibration reference for establishing the laboratory origin and calibrating the motion capture system.

**Figure 2.**
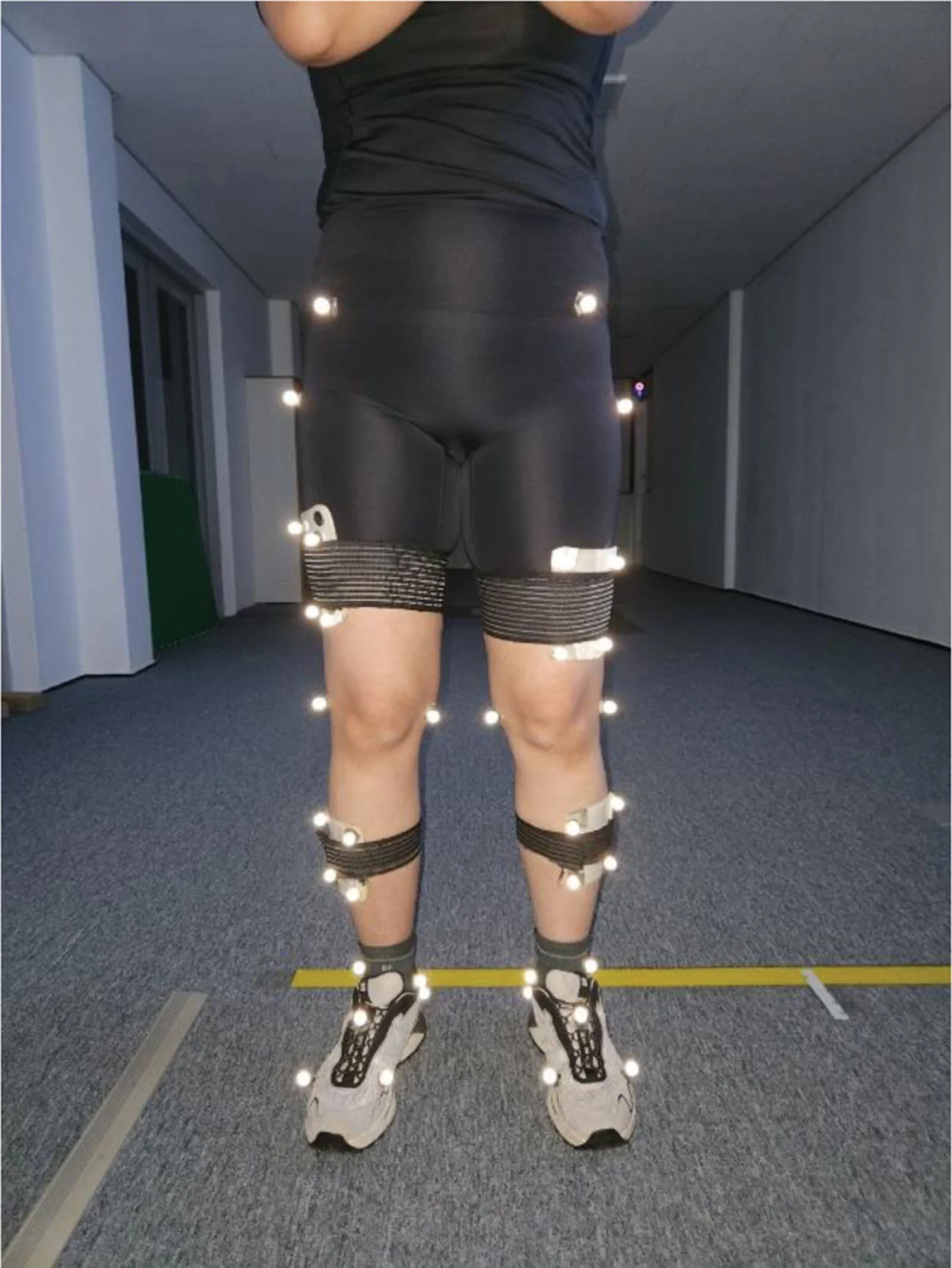
A set of 40 reflective markers positioned on the pelvic segment and lower extremities for the static calibration of the skeletal model and collection of kinematic data.

**Figure 3.**
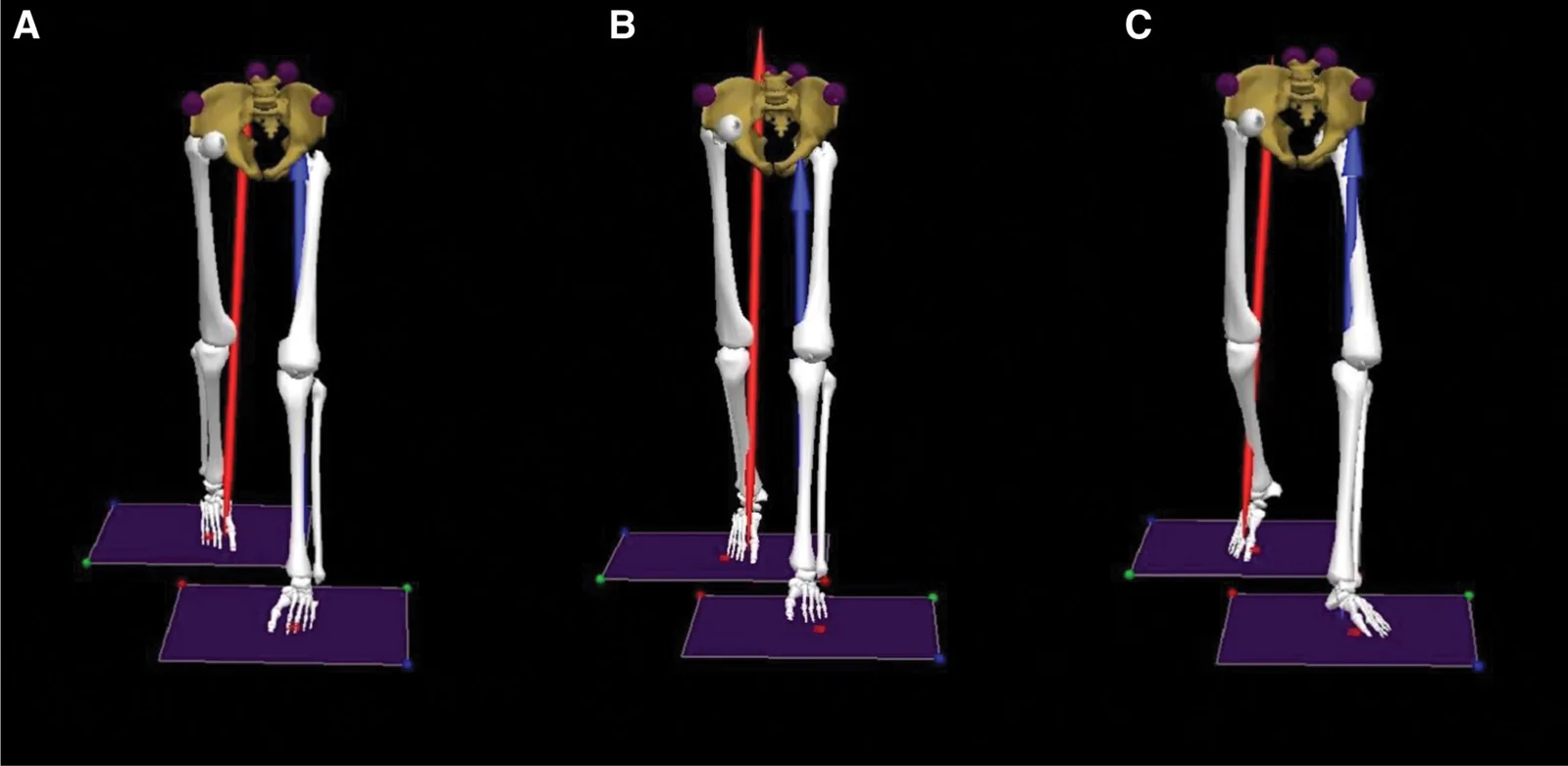
Visual3D representation of a virtual pelvic segment using 3 distinct foot progression angles during walking trials. (A) Maximal internal foot position; (B) neutrally aligned foot position; (C) maximal external foot position. 3D = three-dimensional.

**Figure 4.**
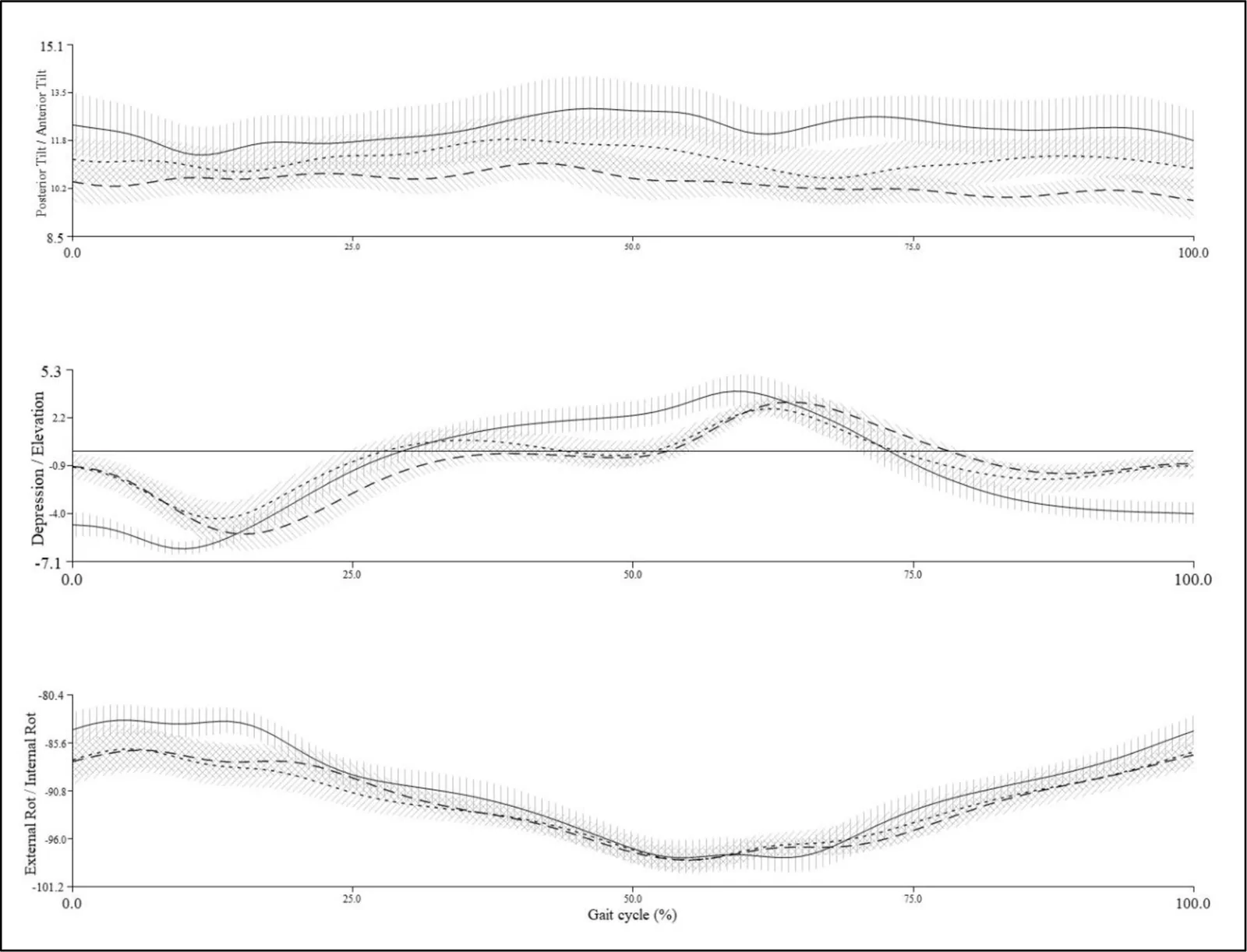
Intraindividual variability in the kinematic range of the pelvis across 3 planes of motion during walking. Average range of pelvic motion derived from 24 repetitive walking trials. Neutrally aligned foot position: solid line with a vertical standard deviation band; maximal internal foot position: dotted line with an upward standard deviation band; maximal external foot position: dashed line with a downward standard deviation band.

A Nexus software program (version 1.8.5, Vicon Inc., Oxford, England) was deployed to integrate data from camera captures and force plates, facilitating the creation of a basic musculoskeletal model for each participant via reflective markers attached to the anatomical landmarks of the joints and segments (Fig. 2).^[4]^ The c3d files generated by the Nexus program were transferred to the Visual3D v6 professional program (C-Motion Inc., Germantown). This facilitated the analysis of all final graphical reports and 3D kinematic pelvic data based on the Calibrated Anatomical System Technique under 3 different walking conditions with FPA modifications (Fig. 3).^[26]^ Kinematic camera capture data were processed using a 4th order Butterworth filter with a cutoff frequency of 6 Hz, while kinetic force platform data were similarly filtered with a 4th order Butterworth filter at a cutoff frequency of 15 Hz.^[26]^ Figure 4 displays examples of 3D pelvic motion during gait trials.

### 2.3. Experimental procedure

This study utilized a randomized crossover design. The experimental protocols included 3 distinct walking trials with FPA modifications: FFP, MIFP, and MEFP. To maintain consistent foot rotation angles for each FPA modification, the angle during unguided walking was set as the baseline FFP condition. The MIFP and MEFP conditions were then established by adding or subtracting approximately 20° from the baseline FFP angle.^[27]^ Additionally, tape guidelines were placed along the walking path to ensure consistent walking velocity and step width, allowing participants to better gauge the extent of FPA rotation achieved during their walking practice.^[4,28]^

To obtain kinematic data on pelvic range of motion (ROM) during gait analysis, 40 reflective markers (1.4 cm) were attached to the pelvic segment, bilateral lower limbs, feet, malleoli, femoral condyles, greater trochanters, anterior superior iliac spines, and posterior superior iliac spines. Each cluster of 4 markers was secured with Velcro to both the calf and thigh segments, following the 6 degrees of freedom model.^[26]^ After attaching reflective markers, an initial static capture was performed using a camera while participants stood to create a template model. This model was then used to analyze dynamic pelvic ROM values under FPA-modified gait conditions. For each FPA-modified walking condition, participants were instructed to walk along a 10 m pathway at a freely chosen speed. A total of 8 successful walking trials were conducted for each FPA modification, with kinematic data from the pelvic segment collected for final analysis. If a walking trial was incomplete due to marker loss or foot position errors in an FPA condition, an additional trial was conducted. The sequence of each FPA condition was randomly determined by a dice throw.

### 2.4. Statistical analysis

To compare pelvic ROM during each FPA modification walking trial, maximal ROM variables in each 3D motion plane were analyzed. Six maximal pelvic ROM variables were assessed: anterior tilting and external rotation occurring during 30% to 60% of the gait cycle; posterior tilting and elevation during 50% to 80% of the gait cycle; and depression and internal rotation during 0% to 30% of the gait cycle. A repeated-measures analysis of variance (ANOVA) with Bonferroni adjustment was used to compare the kinematic data of the pelvic segment based on FPA modification conditions and lower extremity sides. If the main effect (FPA condition or extremity side) was significant, post hoc tests were conducted to confirm pairwise comparisons based on the ANOVA results. The Kolmogorov–Smirnov test was applied to verify the normal distribution of the pelvic ROM data. All analyses were performed using SPSS version 29.0 (IBM Corp., Armonk), and differences were considered significant at an α = 0.05 level.

## 3. Results

Mauchly assumption of sphericity was satisfied for all maximal pelvic ROM variables required for repeated measures ANOVA analysis. Significant differences were observed in some maximal pelvic ROM variables among the FPA modification conditions during gait (Table 1). The maximal elevation (*F*_(2,72)_ = 3.773, *P* = .041), maximal depression (*F*_(2,72)_ = 6.904, *P* = .007), and total ROM in the frontal plane (*F*_(2,72)_ = 4.007, *P* = .036) demonstrated statistically significant differences under varying FPA modification conditions (Table 1). Additionally, maximal internal rotation (*F*_(2,72)_ = 14.627, *P* = .0001) and maximal external rotation (*F*_(2,72)_ = 7.660, *P* = .005) also showed significant differences under different FPA walking conditions (Table 1). However, no significant differences in any maximal pelvic ROM values were observed between lower limb sides (*P* > .05) (Table 1). Furthermore, no interactive effects were found between the FPA modification conditions and lower limb sides in any of the maximal pelvic ROM variables (*P* > .05) (Table 1).

**Table 1:** Comparison of three-dimensional pelvic range of motion by FPA gait conditions and lower leg sides in the stance phase during gait (repeated measures analysis of variance, N = 37).

Maximal pelvic motion	Level	*F*	*P*-value
Anterior tilting	Gait conditions	1.851	.181
	Leg sides	0.273	.755
	Conditions × sides	1.444	.240
Posterior tilting	Gait conditions	0.469	.488
	Leg sides	1.001	.361
	Conditions × sides	1.159	.293
Total range in sagittal plane	Gait conditions	1.093	.355
	Leg sides	0.804	.407
	Conditions × sides	1.110	.311
Elevation	Gait conditions	3.773	.041*
	Leg sides	1.244	.264
	Conditions × sides	1.378	.277
Depression	Gait conditions	6.904	.007*
	Leg sides	1.440	.253
	Conditions × sides	1.771	.196
Total range in frontal plane	Gait conditions	4.007	.036*
	Leg sides	1.913	.160
	Conditions × sides	2.106	.122
Internal rotation	Gait conditions	14.627	.000*
	Leg sides	0.994	.374
	Conditions × sides	1.408	.274
External rotation	Gait conditions	7.660	.005*
	Leg sides	0.988	.492
	Conditions × sides	0.950	.324
Total range in transverse plane	Gait conditions	1.897	.144
	Leg sides	0.718	.400
	Conditions × sides	1.852	.159

FPA = foot progression angle.

* *P* < .05.

Table 2 presents the mean values of maximal pelvic ROM under 3 different FPA conditions during gait. Under the MIFP walking condition, participants exhibited less maximal depression, reduced total frontal pelvic motion, decreased maximal external rotation, and increased maximal internal rotation compared with the FFP walking condition (Table 2). The application of the MEFP condition resulted in significantly less internal rotation and increased external rotation values in pelvic ROM compared to the FFP walking condition (Table 2). No significant differences in anterior and posterior tilting variables were noted among the 3 FPA conditions, WFO and SFO (*P* > .05) (Table 2).

**Table 2:** Average pelvic three-dimensional motion values across 3 different foot progression angle conditions during gait (repeated measures analysis of variance with Bonferroni correction, N = 37).

Maximal pelvic motion (°)	MIFP	FFP	MEFP
Anterior tilting	1.42 (0.93)	1.42 (0.80)	1.57 (0.84)
Posterior tilting	1.29 (0.63)	1.44 (0.71)	1.31 (0.77)
Total range in sagittal motion	2.71 (1.22)	2.86 (0.82)	2.88 (0.99)
Elevation	5.42 (2.62)	5.28 (2.55)	4.95 (1.92)†
Depression	3.27 (1.19)*	3.90 (1.33)	3.95 (1.42)†
Total range in frontal motion	8.69 (2.77)*	9.18 (2.24)	8.90 (2.13)
Internal rotation	5.43 (1.46)*	2.59 (1.15)	0.54 (0.93)*^,^†
External rotation	9.04 (3.24)*	12.37 (3.84)	15.01 (4.55)*^,^†
Total range in transverse motion	14.47 (3.88)	14.96 (4.03)	15.55 (3.72)

Data were presented as mean (standard deviation).

FFP = free foot position, MEFP = maximal external foot position, MIFP = maximal internal foot position.

* Significant difference compare to the FFP condition, *P* < .05.

† Significant difference compare to MIFP conditions, *P* < .05.

## 4. Discussion

Understanding the biomechanical characteristics of the segments and joints of the lower limbs in patients with knee OA in response to various FPA modification conditions during gait is critical.^[4,12,15,18,28]^ Prior research assessing the efficacy of FPA modifications as a therapeutic gait intervention for medial compartment knee OA mainly examined changes in the kinetic KAMs of the knee joint itself^[4,12,15,18–20]^ and focused on kinematic dependent variables affecting the hip, knee, and ankle joints.^[21,28–30]^ Consequently, this study aimed to investigate the impact of toe-in and toe-out FPA modifications on 3D pelvic kinematic characteristics during free-speed walking trials in individuals with knee OA.

The strength of this study lies in its utilization of a 3D motion analysis system to measure pelvic kinematic data during FPA-modified gait, widely acknowledged as highly reliable within the field of biomechanics.^[31]^ Furthermore, this study is the first to assess 3D pelvic kinematic variables across various FPA modification walking trials in individuals with knee OA. The findings indicated that the ROM in pelvic depression significantly decreased at the onset of the gait cycle, while external rotation increased in the MIFP walking trial compared to the MEFP and FFP conditions. Conversely, the maximal ROM for pelvic internal rotation was greater in the MIFP walking condition than in the FFP and MEFP conditions. However, no changes were observed in the anterior and posterior tilting ROM of the pelvic segment in the sagittal plane among the 3 FPA walking conditions. Few previous studies provide a direct comparison with the pelvic movement results presented in our study for each FPA walking application.

A prior study evaluated kinematic pelvic motions under 3 different foot position angles (toe-in, neutral, and toe-out) while participants were in a standing posture.^[30]^ Similar to our findings, the reported pelvic movement angles under the 3 FPA conditions in the standing posture, as described by Haba and Iwatsuki,^[30]^ predominantly showed changes in the transverse plane, while no alterations in sagittal pelvic motions were noted. Thus, compared to FFP gait, the adherence of pelvic movement to toe-in or toe-out FPA changes was primarily related to rotational kinematic movement.

Conversely, prior studies investigating the elevation and depression movements of the pelvic segment did not completely align with our results. Notably, previous research found no difference between the toe-in FPA condition and the neutral FPA condition regarding the maximal pelvic depression angle, unlike in this study. This discrepancy can be attributed to differences in measurement procedures and research methodologies, including the clinical characteristics of the participants (healthy adults vs individuals with knee OA) and the posture during FPA application (standing vs walking).

Our results revealed more pronounced changes in pelvic elevation and depression ROM variables between the MIFP and MEFP modification conditions. However, no differences in overall pelvic ROM in the frontal motion plane were observed. Additionally, disparities in the ROM values for pelvic internal and external rotations were evident under each MIFP and MEFP walking condition, but no notable differences in total transverse pelvic motions between these 2 FPA modification conditions were found. This variability likely results from the FPA gait trials employing contrasting foot positions (toe-in vs toe-out), affecting overall pelvic frontal and transverse motions.

Consequently, the clinical intervention of toe-in or toe-out FPA modification during walking in individuals with knee OA did not appear to significantly impact overall pelvic movements across 3D motion planes. The total maximum ROM of the pelvic segment in each 3D motion plane was comparable among the 3 FPA modification interventions. However, excessive pelvic internal rotation was observed when MIFP was applied, while excessive pelvic external rotation occurred with the MEFP application. This phenomenon likely represents an excessive rotational response of the pelvic segment to the artificially induced rotational gait of the lower extremities prompted by toe-in or toe-out walking. Pelvic instability, resulting in abnormally increased movement of the pelvic segment during walking, is recognized as a contributing factor to LBP.^[31–33]^

The clinical implications of this study are summarized as follows: toe-in or toe-out FPA modification walking trials, although recommended to reduce peak KAMs and alleviate knee pain in certain groups with knee OA, may not be significantly effective.^[6]^ FPA modification walking intervention cannot completely eliminate the risk of developing musculoskeletal complications associated with knee osteoarthritis, such as LBP and pelvic issues.^[34,35]^ LBP is often comorbid in individuals with knee OA, with a prevalence of 57.4% among knee OA patients.^[35]^ Although the etiology, clinical characteristics, and mechanisms underlying LBP as a complication of knee OA remain elusive, evidence supports an interrelationship between knee OA and LBP.^[25]^ Structural or functional factors that may contribute to complications in LBP for individuals with knee OA, such as abnormal repetitive postures and movements, should be carefully evaluated in the management of knee OA.

This study had several limitations. Most participants had mild to moderate knee OA and did not experience knee joint pain that hindered walking, which limits the generalizability of these findings to patients with severe knee OA. Additionally, the study did not include a clinical evaluation of other musculoskeletal comorbidities, such as LBP, which could help clarify the 3D pelvic mobility characteristics influenced by FPA modification interventions. Further research is needed to investigate the relationship between the severity of knee OA and the biomechanical characteristics of the pelvic segment in patients with both knee OA and LBP through various clinical interventions.

## 5. Conclusions

The study revealed that a 20° toe-in or toe-out FPA modification gait induced excessive pelvic internal or external rotation compared to the neutral FPA gait. This response is deemed an excessive rotational adaptation of the pelvic segment to compensate for the lower extremities’ rotation due to the FPA gait interventions. Abnormal movements of the pelvic segment during gait may contribute to the development and exacerbation of LBP, which is a common comorbidity in patients with knee OA. Therefore, while implementing the toe-in or toe-out FPA modification gait in individuals with knee OA, known to reduce peak KAMs, the presence of additional musculoskeletal dysfunctions, such as back pain and their clinical implications, should be considered.

## Acknowledgments

We appreciate the contributions of all participants, funds that assisted in the work.

## Author contributions

**Conceptualization:** Yongwook Kim.

**Data curation:** Yongwook Kim.

**Funding acquisition:** Yongwook Kim.

**Methodology:** Yongwook Kim.

**Project administration:** Yongwook Kim.

**Resources:** Yongwook Kim.

**Software:** Yongwook Kim.

**Writing – original draft:** Yongwook Kim.

**Writing – review & editing:** Yongwook Kim.
